# Where Have the Dead Gone?

**DOI:** 10.3389/fmed.2022.837287

**Published:** 2022-03-16

**Authors:** Michael Günther, Falk Mörl, Robert Rockenfeller

**Affiliations:** ^1^Computational Biophysics and Biorobotics, Institute for Modelling and Simulation of Biomechanical Systems, Universität Stuttgart, Stuttgart, Germany; ^2^Friedrich–Schiller–Universität, Jena, Germany; ^3^Forschungsgesellschaft für Angewandte Systemsicherheit und Arbeitsmedizin mbH, AG Biomechanik & Ergonomie, Erfurt, Germany; ^4^Mathematisches Institut, Universität Koblenz-Landau, Koblenz, Germany

**Keywords:** pivotal clinical trial, SARS-CoV-2, mortality, prognosis, vaccine

Data published from pivotal clinical trials contain the key information on *efficacy* and *safety* of newly developed drugs. With the magnified public awareness focused around the world on the SARS-CoV-2 existence, the authors, albeit medical laymen though ardent scientists, could likewise not escape being prodded to research data published in the virological and epidemiological fields. This commentary is a consequence of our ruminating what the trials' published *safety* data tell us.

**To Say It Outright:** We do not understand why plain and *important* data informing of vaccination safety seem to be unpublished as a rule, namely, the plain count of how many persons had died in each trial group during the time interval from receiving the first dose of a vaccine (or placebo, respectively) until the end of the follow-up period (the monitoring interval). The high scientific value of such raw death counts comes, in particular, from not depending on any selection criterion applied to the collected data. The counts should be given for each randomized trial group, usually, the *placebo*-treated and the *vaccinated* one. These overall death counts, first of all for the *placebo* group, can then be compared with a number that is prognosticated from (i) the absolute number of persons within each age cohort the groups consist of, (ii) known annual mortality rates of the age cohorts, and (iii) the time interval between first dose reception and follow-up termination. In a second step, the prognosticated number of deaths and the dead counted in the *placebo* group can be compared with the dead counted in the *vaccinated* group. We here apply the prognosis method to the first three pivotal clinical trials on SARS-CoV-2 vaccines, BNT162b2 (1), mRNA-1273 (2), and Ad26.COV2.S (3), with the method certainly being applicable to any clinical fatality examination or safety trial as, e.g., most recent ones (4, 5). The comparisons presented here are facilitated by the three trials probed having compared groups that all consist of nearly matching age cohorts.

Analysing (1), we can calculate from their Table 1 and their Supplementary Table S4 that the cohort distributions of the two trial groups reflected in their Supplementary Table S3, which consist of each about 21,620 persons, can be divided into 12,502 persons in the age cohort [16-55]y (“y” symbolizing “year,”) 4,702 persons in the cohort [56–65]y, and 4,416 persons in their cohort [>65]y. We estimated annual mortality rates of these cohorts by taking the German population data (6) as a proxy: 0.15% for [16–55]y, 0.7% for [56–65]y, and 1.5% for [66–75]y. We prognosticate (“w” symbolizing “week”)


$$\begin{array}{l} \;21620\cdot \frac{11\text{w}}{52\text{w}}\cdot \underset{}{\underset{︸}{\left(0.0015\cdot \frac{12502}{21620}+0.007\cdot \frac{4702}{21620}+0.015\cdot \frac{4416}{21620}\right)}}\\ \approx 21620\cdot \frac{11\text{w}}{52\text{w}}\cdot \underset{}{\underset{︸}{\left(0.0015\cdot 0.58+0.007\cdot 0.22+0.015\cdot 0.20\right)}}\\ \approx 21620\cdot \frac{11\text{w}}{52\text{w}}\cdot \underset{}{\underset{︸}{\left(0.00087+0.00154+0.003\right)}}\;\approx \;21620\cdot \frac{11\text{w}}{52\text{w}}\cdot \underset{}{\underset{︸}{0.0054}}\\ \approx 4+7+14\\ =25 \end{array}$$

persons that were expected to die in the (1) *placebo* group during the monitored time interval of three (between first and second dose) plus eight weeks (follow-up after second dose). This is nearly an order of magnitude higher than the 4 dead given in (1, their Supplementary Table S3), whilst this Table apparently reflects “events” in a subset of only 2,638 persons. The number of just 2 dead in a subset of 5,770 *vaccinated* seems then even more inconsistent.

In the above calculation (prognosis), we have marked by under-bracing the study-specific overall annual mortality rate, which arises (see first row of the calculation) from the weighted sum of the three cohort-specific annual mortality rates (each the first factor in a summand), with their weights (each the second factor in a summand) being the study-specific fractions of the cohorts. Related to the trial study (1) investigated here, the prognosis method requires seven parameters to be known: the monitoring interval (eleven weeks) plus both the numbers of persons and the annual mortality rates for each age cohort taken into account (three). The uncertainty of the prognosticated number of deaths is calculated in the section “**Estimating the uncertainty of the prognosticated number of deaths**” appended to this communication. In short, assuming age cohort weighting in the trials to be uncertain among oldest and youngest by 10%, while knowing how the cohorts' mortality rates vary among the recruiting regions for the trial groups, we calculate the *minimum estimate* of the prognosticated number of deaths to be 25% lower than the 25 deaths calculated above on the basis of German annual mortality rate values, thus, 19 deaths in the trial study (1) just investigated.

In continuation of Polack et al. (1), interestingly, the authors of (7) finally counted 15 (*vaccinated*) and 14 (*placebo*) dead during 11 weeks, then evidently giving numbers for complete trial groups (see their Supplementary Table S4; 21,920 persons). If our German-based estimate (25 deaths in 21,620 persons) is assumed to be the expected value of a binomial probability distribution then the corresponding standard deviation is quite exactly 5. A corresponding binomial test reveals that the 14 dead in the *placebo* group are significantly different from prognosticated 25 deaths, at a *p*-value of 0.012. Hence, counting just 14 dead in Thomas et al. (7) is already utterly unlikely to be explainable by chance; and the four dead reported in Polack et al. (1) are an entirely impossible count, which can only reflect some preliminary data analysis. In stark disaccord, the data reported in Polack et al. (1) should without doubt stand on their own and not rely on additional publications, as this was a public dissemination of both safety and efficacy probed by a pivotal vaccine trial. Publishing another, later (6-month) safety data set as in Thomas et al. (7), or even secondary reports like, e.g., by the USA's “Food and Drug Administration,” should not be required when disseminating a primary endpoint assessment of safety (mortality).

Due to the age cohort distributions nearly matching in the three pivotal clinical trial studies (1–3) investigated here (i.e., the overall annual mortality rate is assumed to have the same value 0.0054, within the uncertainty estimated below), the 25 deaths prognosticated for (1) can be easily scaled to the trial groups in (2, their Supplementary Table S8), which consist of each about 15,200 persons. As the monitored interval was four (between first and second dose) plus four weeks (follow-up after second dose), the prognosis becomes $25\cdot \frac{15200}{21620}\cdot \frac{8\text{w}}{11\text{w}}\approx 13$ deaths during eight weeks. Their data (2, their Supplementary Table S8) tell 3 dead in an apparent “event” subset of 3,277 persons within the *placebo* group, and 2 dead in 3,632 “event”-attributed *vaccinated* persons.

Further astonishing numbers can be found, if the prognosis is applied to the trial groups in Sadoff et al. (3): The prognosticated deaths can likewise be easily calculated by scaling, for a trial group now consisting of about 21,900 persons. We calculate $25\cdot \frac{21900}{21620}\cdot \frac{4\text{w}}{11\text{w}}\approx 9$ deaths, whereas 16 dead are documented in (3, their Supplementary Table S7) for the “event” subset of the *placebo* group, with half of them attributed to having died of CoViD-19. In the *vaccinated* group then again, only 3 died during the four weeks after the single dose treatment, which is again much lower a number then prognosticated. The “event” subsets seem to have consisted of 3,380 and 3,356 persons, respectively.

As our conclusion, in perfect line with a recent scientific request (8) for raw trial data, we urgently ask researchers carrying out pivotal clinical trials: Please, publish the *entire* number of deaths having occurred in each *complete* trial group during the monitoring interval! They are the most meaningful and reliable data recorded in these trials, as they lack any potential interpretative bias by conceivably applying selection criteria after recording. Accordingly, giving the full and raw number of fatalities within each the *placebo-*treated and the *vaccinated* trial group during a monitoring interval (identical for all treated persons) after the first treatment is critical to assess the efficacy *and* safety of the vaccines.

Failing to properly measure and report mortality, a primary endpoint, is inexcusable. Unconditional opening of the scientific raw trial data (9), which are claimed to be privately “owned” yet decisively shape public policy, is a remedy for such failure.

## Estimating the Uncertainty of the Prognosticated Number of Deaths

The formula given above enables calculating prognoses for any comparable study in the literature. Here, we provide information of *how uncertain* the prognosticated death values are, given empirically known uncertainties of the input parameters for the prognosis. More exactly, we provide an absolute *minimum estimate* of our prognosis and estimates of how much a prognosticated value *goes up* if US, Brazilian, or South-African mortality rates contribute, rather than German ones. We start with explaining how we distinguished, by inference from supplementary data, *three* age cohorts in Polack et al. (1) study, although only *two* are explicitly set out in their main paper.

Table 1 in Polack et al. (1) (“main safety population:” about 18,850 persons per group) lists an age resolution of two cohorts, [16–55]y and [>55]y, whereas their Supplementary Table S3 lists numbers of persons in the overlapping cohorts [16–65]y and [>65]y, with these groups (about 18,250 persons) being slightly smaller due to having picked out about 600 persons “infected prior to dose 2.” Accepting a corresponding inaccuracy of maximally 150 persons, which includes assuming the persons removed to be approximately in proportion to the cohort fractions, one can estimate the number of persons in the overlapping cohort part [56–65]y (4,702) as the difference (about 4,100) between the number in [>55]y from (1, their Supplementary Table 1) and the number in [>65]y from (1, their Supplementary Table S3), and this difference then scaled by the ratio of trial to “main safety” group numbers ($\frac{21620}{18850}$).

The trial groups in Polack et al. (1) consisted of 76.7% persons from the USA, 15.3% from Argentina, 6.1% from Brazil, and 2% from South Africa. The groups in Baden et al. (2) consisted of USA citizens by 100%. In Sadoff et al. (3), the groups consisted of 44.1% persons from the USA, 16.6% from Brazil, 15% from South Africa, and 24.3% from other Latin-American countries. The USA's annual mortality rates (10) of the cohorts [15–54]y, [55–64]y, and [65–74]y are, 0.18, 0.88, and 2,3%, respectively, i.e., the USA-based values are in general at least 20% *higher* than the German-based ones (0.15, 0.7, and 1.5%), which would accordingly increase the overall mortality rate from 0.0054 (German-based) to about 0.0065 (USA-based). In Brazil (11, 12), we find 0.23, 0.98, and 2,45%, respectively, which are nearly 50% *higher* than in Germany. In South Africa (13, 14), we find 0.55, 1.82, and 3,13%, respectively, which are all more than 100% *higher* than in Germany.

Thus, regarding mortality rates alone, using German values as a proxy minimizes the number of prognosticated deaths.

To identify an absolute *minimum estimate* of the number of prognosticated deaths, we first of all hypothesize as an extreme that the persons in the cohort [66–75]y of a trial group may only be half as many, i.e., we consider another source of uncertainty of the study-specific cohort distribution, namely, we assume a study-specific [66–75]y fraction value of 0.10 rather than 0.20, together with adding all these persons to the [16–55]y cohort. As a result, the number of prognosticated deaths would be 20% lower than any prognosis calculation based on German mortality data. Next, the number of persons in a trial group is well-defined, and the monitored interval is uncertain by less than half a week. Accordingly, in the above prognosis calculation, the whole multiplier (product of the number of persons in a group and the monitored weeks, divided by the weeks in a year) to the overall mortality rate is uncertain by maximally 1%; correspondingly, this source of uncertainty is almost insignificant. Eventually, some further source of uncertainty is inherent to the parameter values assumed for the mortality rates. As the monitoring interval consists of the order of ten weeks, and reflects the epidemiological conditions at specific places and during a specific period in time, the natural fluctuations of the weekly mortality rates may be taken into account. In Germany, about 19,200 all-cause deaths currently occur per week, with an empirically determined long-term standard deviation of about 250 persons [combined (6) and (15)]. Thus, a weekly mortality rate is uncertain on the level of about 1.3% .

All in all, the absolute *minimum estimate* of the number of prognosticated deaths is 20% (cohort fraction uncertainty) plus 1% (monitoring interval uncertainty) plus 4% (approximately three times the uncertainty of weekly mortality rates), that is, 25% below the number of deaths that is prognosticated on the basis of German annual mortality rate values.

## Author Contributions

MG did the calculations. All three authors contributed to writing the words and revising the text.

## Funding

The authors have not been funded while writing this commentary; publication costs were covered by the Open Access Fund of the University of Koblenz-Landau.

## Conflict of Interest

The authors declare that the research was conducted in the absence of any commercial or financial relationships that could be construed as a potential conflict of interest.

## Publisher's Note

All claims expressed in this article are solely those of the authors and do not necessarily represent those of their affiliated organizations, or those of the publisher, the editors and the reviewers. Any product that may be evaluated in this article, or claim that may be made by its manufacturer, is not guaranteed or endorsed by the publisher.
