# Corrigendum: Where have the dead gone?

**DOI:** 10.3389/fmed.2023.1310746

**Published:** 2023-10-30

**Authors:** Michael Günther, Falk Mörl, Robert Rockenfeller

**Affiliations:** ^1^Computational Biophysics and Biorobotics, Institute for Modelling and Simulation of Biomechanical Systems, Universität Stuttgart, Stuttgart, Germany; ^2^Friedrich–Schiller–Universität, Jena, Germany; ^3^Forschungsgesellschaft für Angewandte Systemsicherheit und Arbeitsmedizin mbH, AG Biomechanik & Ergonomie, Erfurt, Germany; ^4^Mathematisches Institut, Universität Koblenz-Landau, Koblenz, Germany

**Keywords:** pivotal clinical trial, SARS-CoV-2, mortality, prognosis, vaccine

In the published article, there was an error. At one text position, the level of significance stated as “*p*-value of 0.0012” is incorrect, given too high by exactly an order of magnitude.

A correction has been made to Paragraph 5. This sentence previously stated:

“In continuation of Polack et al. (1), interestingly, the authors of (7) finally counted 15 (*vaccinated*) and 14 (*placebo*) dead during eleven weeks, then evidently giving numbers for complete trial groups (see their **Supplementary Table S4**; 21,920 persons). If our German-based estimate (25 deaths in 21,620 persons) is assumed to be the expected value of a binomial probability distribution then the corresponding standard deviation is quite exactly 5. A corresponding binomial test reveals that the 14 dead in the *placebo* group are significantly different from prognosticated 25 deaths, at a *p*-value of 0.0012. Hence, counting just 14 dead in Thomas et al. (7) is already utterly unlikely to be explainable by chance; and the 4 dead reported in Polack et al. (1) are an entirely impossible count, which can only reflect some preliminary data analysis. In stark disaccord, the data reported in Polack et al. (1) should without doubt stand on their own and not rely on additional publications, as this was a public dissemination of both safety and efficacy probed by a pivotal vaccine trial. Publishing another, later (6-month) safety data set as in Thomas et al. (7), or even secondary reports like, e.g., by the USA's “Food and Drug Administration,” should not be required when disseminating a primary endpoint assessment of safety (mortality).”

The corrected sentence appears below:

“In continuation of Polack et al. (1), interestingly, the authors of (7) finally counted 15 (*vaccinated*) and 14 (*placebo*) dead during 11 weeks, then evidently giving numbers for complete trial groups (see their **Supplementary Table S4**; 21,920 persons). If our German-based estimate (25 deaths in 21,620 persons) is assumed to be the expected value of a binomial probability distribution then the corresponding standard deviation is quite exactly 5. A corresponding binomial test reveals that the 14 dead in the *placebo* group are significantly different from prognosticated 25 deaths, at a *p*-value of 0.012. Hence, counting just 14 dead in Thomas et al. (7) is already utterly unlikely to be explainable by chance; and the four dead reported in Polack et al. (1) are an entirely impossible count, which can only reflect some preliminary data analysis. In stark disaccord, the data reported in Polack et al. (1) should without doubt stand on their own and not rely on additional publications, as this was a public dissemination of both safety and efficacy probed by a pivotal vaccine trial. Publishing another, later (6-month) safety data set as in Thomas et al. (7), or even secondary reports like, e.g., by the USA's “Food and Drug Administration,” should not be required when disseminating a primary endpoint assessment of safety (mortality).”

The authors apologize for this error and state that this does not change the scientific conclusions of the article in any way. The original article has been updated.

